# A Novel Point-of-care Ultrasound Curriculum for Air Critical Care Personnel

**DOI:** 10.5811/westjem.2022.12.57599

**Published:** 2023-01-09

**Authors:** Laurel O’Connor, Matthew Beth-Urhoy, Stephen Allegra, Andrew Dowd, Alexandra Nordberg, Timothy Boardman, Timothy Gleeson, Robert Lindsay

**Affiliations:** * University of Massachusetts Chan Medical School, Department of Emergency Medicine, Worcester, Massachusetts; † University of Massachusetts Chan Medical School, Department of Radiology, Worcester, Massachusetts

## Abstract

**Introduction:**

Point-of care-ultrasound (POCUS) has become ubiquitous in emergency medicine practice for the management of emergent pathophysiology. There is growing interest in its potential as a diagnostic tool in the prehospital setting. Few studies have examined the feasibility or efficacy of curricula targeted at teaching POCUS to prehospital personnel. Our objective in this study was to investigate a curriculum for the extended focused assessment with sonography in trauma (eFAST) exam in helicopter emergency medical services (HEMS) crews.

**Methods:**

This was a pre/post intervention study of HEMS personnel at a tertiary care center. Subjects were administered a pre-intervention written test and an observed structured clinical evaluation (OSCE). Subsequently, they participated in an educational intervention intended to impart proficiency in performing the eFAST. Subjects underwent post-intervention written exams and OSCEs. We analyzed pre- and post-intervention test performance along with the number and quality of practice ultrasound examinations achieved.

**Results:**

Sixteen subjects were enrolled (62.5% male, mean age 44.1). After undergoing the intervention, the mean written test score increased 22.1% (t=3.41; P <0.001) and the mean OSCE score increased by 64.5% (t=6.87, P <0.001). All subjects met “passing” criteria for the written test and OSCE on their post-intervention attempt. Subjects accomplished a mean of 21.1 clinically interpretable eFAST sonographs. Most subjects reported the curriculum was useful (90.1%) and that they would incorporate this skill into clinical practice (90.1%).

**Conclusion:**

A targeted POCUS curriculum was feasible and effective in establishing clinical proficiency in HEMS crews for performing and interpreting the eFAST exam.

## INTRODUCTION

Ultrasound-mediated imaging plays an essential role in acute patient care. It is a unique imaging modality in that it is non-invasive, involves no radiation, and can capture dynamic processes rather than only static images.^1^ In the past 20 years, point-of-care-ultrasound (POCUS) has become ubiquitous in emergency medicine practice for the diagnosis and management of emergent pathophysiology as well as to facilitate common emergent procedures.^2^

The use of POCUS is no longer limited to brick-and-mortar clinical spaces. New advancements in ultrasound technology have yielded the development of machines small enough to fit in a medical aircraft or vehicle without compromising image quality, thereby granting ultrasound access to areas previously constricted by free space, including medical transport helicopters and ambulances. There is a historical precedent for out-of-hospital POCUS: It has been used by ground and air emergency services by the United States military and in parts of Europe as early as the late 1990s for detection of heart, lung, and vascular emergencies.^3^ Although the image quality was not as high as that generated by the cart-based machines used in hospitals and clinics, it was possible to detect chest and abdominal abnormalities.^4^ These deviations in image quality have improved due to breakthroughs in imaging technology, paving the way for more mainstream utilization in the prehospital environment.^5^

In the hyper-acute management of critical care patients there are two major diagnostic uses for POCUS that direct the care of time-sensitive and life-threatening pathologies. First, POCUS is highly sensitive for clinically significant pneumothorax.^6^,^7^ While pneumothorax can also be detected by other means including physical exam, a comprehensive exam may prove challenging during helicopter transport of patients because of environmental noise and movement. A POCUS exam can diagnose or confirm clinically suspect pneumothorax in real time. Intra-abdominal bleeding can also be detected with POCUS. The extended focused assessment with sonography for trauma (eFAST) exam requires images of the lungs for pneumothorax as well as left upper quadrant, right upper quadrant, cardiac, and pelvic views.^7^ The eFAST exam can be applied during flight if an ultrasound machine is present in the helicopter. Given that many critical care services now carry blood products, identifying active hemorrhage with ultrasound may help optimize the use of such resources. It will also allow crews to relay critical information to receiving hospitals, direct destination choice, and decrease the time from arrival at the hospital to transfer to an operating room for definitive management.

Ultrasound technology has the potential to improve the quality of patient care during helicopter transport by facilitating the diagnosis and management of life-threatening conditions in the out-of-hospital space. However, it must first be demonstrated that sonography training programs can effectively prepare helicopter emergency medical services (HEMS) crews to master this skillset. There is limited data on the practicality and effectiveness of POCUS training programs for prehospital personnel. Prior feasibility studies have demonstrated modest success in teaching HEMS to perform the eFAST exam, as well as chest wall and thoracic sonography.^8^,^9^,^10^ However, these studies have focused on standard evaluation metrics, such as written tests, and did not measure the clinical interpretability of complete eFAST sonographs achieved by subjects.

We sought to determine whether a novel curriculum would be successful at training HEMS critical care paramedic and nurses to achieve an adequate level of mastery for clinical use of POCUS. The curriculum is innovative first in that it was developed specifically for critical care personnel. The scope and clinical applications used for the didactic were tailored to the out-of-hospital, low-resource environment. Trainees were offered asynchronous learning opportunities necessary for the nonstandard duty hours typical of air critical care services including both expert-supervised and independent, practical learning sessions. Finally, the training was intended to prepare HEMS crews both to excel at standard written and practical evaluations of competency and consistently produce clinically interpretable images. Our objective in this study was to investigate the feasibility and efficacy of this curriculum to render HEMS personnel proficient in the theory and application of the eFAST exam.

**Population Health Research Capsule**

What do we already know about this issue?

*Point of care ultrasound (POCUS) can be used to guide treatment for serious pathologies managed by HEMS crews. There is no validated curriculum for teaching POCUS skills to Helicopter Emergency Medical Services (HEMS) clinicians.*

What was the research question?

*Can an investigational curriculum for POCUS effectively impart proficiency in this skill to HEMS personnel?*

What was the major finding of the study?

*Subjects’ mean written test scores increased by 22%, and mean Objective Structured Clinical Evaluations (OSCE) scores increased by 65%.*

How does this improve population health?

*A POCUS curriculum was effective teaching HEMS providers skills in performing and interpreting the POCUS studies. This skill may be a valuable addition to prehospital care.*

## METHODS

### Study Setting and Participants

The development and implementation of the novel HEMS POCUS curriculum, along with evaluation of its execution and efficacy took place at an urban, tertiary-care center that houses and provides medical direction for a regional air critical care HEMS service. Subjects were recruited via email and in person at scheduled staff meetings. All subjects were licensed critical care paramedics or critical care registered nurses and active crew for the local HEMS service. All personnel were English speaking and provided informed consent to participate. No trainees or orienting subjects on probation were included. No eligible subjects declined to participate. Pregnant women were included in this study; subjects under 18 were excluded. This study was approved by the institutional review board of the affiliate medical school.

### Curriculum Development

Four ultrasound lectures were developed specifically for the training of HEMS crews, adapted from didactics previously used for medical students by the ultrasound division at our institution. All lectures were developed in collaboration between ultrasound and EMS faculty on the study team. Each lecture was one-hour long and focused on foundations of ultrasound concepts and equipment operation, the chest wall ultrasound to evaluate for pneumothorax, the FAST exam to evaluate for free fluid in the abdomen, and a review of clinical applications and image review for the combined eFAST exam. For the structured practical learning components of the curriculum, all sessions were presided over by ultrasound faculty experienced in coaching trainees in performing POCUS studies in clinical settings.

A 30-minute written test for proficiency in the eFAST exam was developed by the ultrasound faculty on the study team, which was comprised of a combination of image-clip interpretation, equipment operation, and clinical scenario questions. Solicited answers were a combination of multiple choice and free responses. For free responses with multiple components, all acceptable answers were decided in advance and partial credit (a single “point”) was awarded for each correct response. The content of the written exam is included in Appendix A. Additionally, a standard rubric for the observed structured clinical evaluation (OSCE) in POCUS eFAST was leveraged as a measurement tool for this study. The OSCE rubric is included as Appendix B.

### Study Procedure

Enrollment of subjects occurred between January 1–March 31, 2021. Collection of data for each subject began on the first day of participation and continued throughout a one-year period during which each subject underwent four formal didactic sessions, three hands-on sessions with standardized patients, and individual-driven, unsupervised practice scanning sessions. This study used the Philips Lumify (Philips Ultrasound, LLC, Bothell, WA) portable handheld ultrasound device identical to the one employed on the aircraft. The system consisted of a single convex (5-2 megahertz) ultrasound transducer and an accompanying tablet with an application to display the images. This device was used for all educational sonograms performed throughout the study period.

All subjects attended the four one-hour didactic learning sessions either synchronously in person or asynchronously via video recording. All didactics were taught by an ultrasound faculty member. All subjects were required to attend didactics before participating in the practical component of the curriculum. After completing all four didactic sessions, subjects attended between two and three, two-hour ultrasound practical sessions during which ultrasound and EMS faculty supervised practice scans on live standardized patients in a simulation setting. These sessions were scheduled every 3–4 months to be evenly spaced out during the year after the didactic curriculum was presented, and subjects were asked to attend at least two formal sessions.

Between supervised practical sessions, subjects were invited to perform educational scans on volunteer patients in the emergency department (ED) either independently or under the supervision of EMS or ultrasound faculty. Subjects were asked to complete a minimum of 25 practice ultrasound scans during the study period but were allowed to attend as many practice scanning sessions as they chose and were permitted to choose between independent practice vs scanning with an expert trainer for all informal sessions. All practice scans were formally scored by a member of the ultrasound faculty, and formative feedback about deficiencies in technique and image quality were provided to subjects after each scanning session as per usual practice in the department for all sonographs obtained throughout the study. Scores and formative feedback were provided via email within 72 hours of a completed practice sessions. The scoring rubric used for the eFAST exams is shown in Appendix C. Subjects were permitted to undergo the post-intervention written exam and OSCE after the completion of the final formal training session, approximately one year after the commencement of the curriculum.

### Measures

This project was a within-subjects pre/post intervention study. We collected and managed all data using *REDCap* electronic data capture tools, version 9.3.0, hosted at University of Massachusetts Chan Medical School.^11^ Subjects’ pre-and post-intervention written test scores were recorded as a percentage of a possible 51 points; a score greater than 70% was considered passing. The pre- and post-written test administered to subjects was identical. Subjects’ pre-and post-intervention OSCE scores were similarly recorded as a percentage of a possible 67 points, with a score greater than 75% considered passing. The passing score for the OSCE and written examination were set before any assessments were administered. Subjects were also scored on a series of OSCE “critical criteria,” any of which, if not met, would render the sonograph clinically non-interpretable.

All practice scans performed by subjects were scored on a standard 1–8 grading rubric by a member of the ultrasound faculty. This grading rubric was already established in the ED where the study was conducted for all clinical and educational studies performed by physicians. A score of 4 or greater indicates that the sonogram is clinically interpretable. Throughout the study period, the number of scans performed per subject, scan scores, and number of scans that met a minimum criterion of being clinically interpretable were recorded. Finally, upon completion of data collection, all subjects were asked to respond to a brief, anonymous acceptability survey rating the usability and value of the curriculum and evaluation tools. A summary of the curriculum development process, using the validated Kern six-step process is depicted in Table 1.^12^

### Analysis

Demographic and personnel proficiency data, along with the number of clinically interpretable scans, scan scores, and acceptability data, were described descriptively. We compared pre-and post-intervention testing scores using paired *t*-tests for continuous variables and chi squares for categorical variables.

## RESULTS

In total, 16 subjects were enrolled in the study, comprised of 10 critical care registered nurses, four critical care paramedics, and two subjects with both certifications. Their demographics are summarized in Table 2. All enrolled subjects successfully completed the initial training curriculum. One subject only completed 24 practice scans while the remaining 15 subjects accomplished the requested minimum of 25 scans. Subjects were prominently male (62.5%) with a mean age of 44.1 years. Most subjects had no previous formal training in ultrasonography. On average, subjects performed 27.5 complete eFAST scans (range 24–32) consisting of both the standard FAST images plus chest wall images.

### Personnel Performance

Two subjects met “passing” criteria on their pre-intervention written test scores, and none met passing criteria on their pre-intervention OSCE. After undergoing the didactic and practical components of the curriculum intervention, the average written test score increased 22.1% (*t*=3.41, *P* <0.001) and the average OSCE score increased by 64.5% (*t*=6.87, *P* <0.001). All subjects met passing criteria for both the written test and OSCE on their first post-intervention attempt. No subject met any of the critical failure criteria during their post-intervention OSCE. Subject proficiency data is summarized in Table 2.

**Figure 1:**
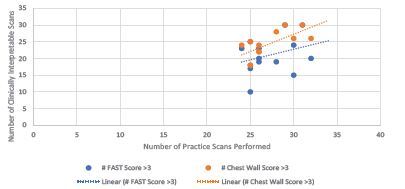
Number of clinically interpretable scans by number of scans performed.

The mean score assigned to subjects’ FAST exams was a 4.73, and the mean score assigned to chest wall scans was 5.64. Subjects accomplished a mean of 21.1 clinically interpretable FAST sonographs and 24.4 chest wall sonographs during their supervised and unsupervised practice sessions. The number of clinically interpretable sonographs performed as a function of total number of practice scans performed is illustrated in Table 3. There was a weak but positive association between increased number of practice scans performed and average score assigned for both the FAST and chest wall sonographs (R=0.44 and R=0.45, respectively). The correlations were stronger between number of scans performed and number of interpretable sonographs produced for both types of scans (R= 0.86 and 0.95, respectively); this is illustrated in Figure 1.

### Acceptability

Subjects’ responses to Likert scale questions pertaining to the acceptability of the novel ultrasound curriculum are summarized in Table 4. Most of the participants either agreed or strongly agreed with positive statements about the acceptability of curriculum including that the lecture component of the curriculum was useful (91%), and that the curriculum was appropriate for HEMS crews (72.7%). They also largely agreed or strongly agreed that after the intervention they could confidently perform a chest wall ultrasound (81.8%) and a FAST ultrasound (81.8%). Overall, 72.7% of subjects agreed or strongly agreed that they gained enough knowledge and experience to comfortably use ultrasound in flight to make clinical decisions as they pertain to lung and FAST ultrasound exams, and 90.1% believed they would incorporate their new ultrasound knowledge and skill in the prehospital setting. Most subjects (72.7%) felt that they would be able to maintain their ultrasound skills after the structured curriculum.

## DISCUSSION

### Personnel Performance

The implementation of a novel curriculum intervention for HEMS personnel was effective in teaching nurse and paramedic subjects with little preexisting knowledge of ultrasound to correctly perform and interpret an eFAST exam in a standardized manner. Subjects were able to successfully participate in a combined didactic and practical learning educational model targeted to their level of medical literacy and subsequently meet standardized benchmarks of proficiency, including consistently producing clinically interpretable sonographs. These results suggest that the curriculum model developed and deployed in this project is efficacious and feasible. Additionally, it suggests that proficiency in limited POCUS skills can be successfully learned and applied by HEMS crews with paramedic and nursing educational backgrounds with a limited, focused intervention. Twenty-four practice scans appeared to be adequate to establish subjects’ ability to produce consistently interpretable sonographs. This is consistent with ultrasound benchmarks established by the American College of Emergency Physicians.^13^

Performing and interpreting POCUS requires a combination of clinical knowledge of anatomy and pathophysiology, procedural skills, including manipulating equipment to optimize image quality, and clinical reasoning skills to interpret and respond appropriately to obtained images in conjunction with other aspects of a patient’s clinical presentation. The improvement in scores from the pre-intervention and post-intervention evaluations suggests that our curriculum imparted adequate education in the theory and application of ultrasound as well as pathophysiology that can be detected with POCUS. There was a notably larger increase in the OSCE scores as compared with the written test. This is likely because while the subjects had some foundational knowledge in the anatomy and pathophysiology required to understand POCUS application as well as the interventions required once pathological conditions are recognized, they had little or no previous experience with operating an ultrasound machine and obtaining and interpreting images. The OSCE evaluation required subjects to operate equipment that was completely unfamiliar to them prior to the initiation of the curriculum, which limited their ability to perform the maneuvers required.

The advantages of the novel curriculum are attributed to the fact that it was created expressly for HEMS personnel. The lectures were developed for non-physicians and taught only by qualified ultrasound faculty with formal training in ultrasound education. The cases used in lecture were tailored to be representative of the pathology frequently encountered by HEMS crews during missions. They could be viewed in real time or asynchronously to be available to all shifts. Additionally, ample time for supervised practice scanning using the actual device planned for use on the aircraft was provided during the study period; and consistent verbal and written feedback, and individualized instruction, was imparted after each practice scan performed. Subjects were offered a variety of options for both supervised and independent scanning sessions that could be performed at the convenience of their schedule; this likely also contributed to the feasibility of the intervention.

### Acceptability

Both the notable increase in written and practical evaluation scores post-intervention, as well as the positive responses on the post-intervention survey suggest that the educational intervention was found to be acceptable and useful by subjects. Subjects generally reported favorable responses to the curriculum, deeming it useful and appropriate for their level of training. They also reported confidence in performing both FAST and chest wall sonographs and felt that they would be able to incorporate their new skills into their existing clinical practice, as well as maintain their skills. These responses further support the feasibility of the curriculum implementation.

## LIMITATIONS

This was a small study performed with a single group of HEMS personnel. There was a narrow range in the number of scans performed and scores achieved. An important subset of subjects had prior exposure to ultrasound education, which could have hastened the establishment of their proficiency in performing the eFAST scans. Since the researchers and evaluators were not blinded to the educational curriculum, observational biases may have occurred during the grading of the OSCEs toward a more favorable outcome. There was also likely some variability in the grading between different assessors. The curriculum was designed to allow subjects to participate asynchronously and perform both supervised and unsupervised practice ultrasounds when feasible for their schedule. However, this may have resulted in a lack of standardization of how many of their scored ultrasounds were achieved with the assistance of a trainer vs independently.

All practice scans were performed in a simulation center or ED; therefore, environmental conditions such as the movement in an aircraft, variations in lighting, performing scans in a helmet and visor, and ability of the patient to cooperate were not considered. Such variations may impact the ability of subjects to practice scans in their usual clinical environment. Additionally, we investigated only a single training strategy, and we did not compare its efficacy to that of other curriculums. Finally, it is possible that there is a more efficient or shorter curriculum design that would impart the same level of proficiency as that elicited by the intervention studied in this project; measuring the minimum amount of time and practice sonographs to establish clinical competency was beyond the scope of this study.

## CONCLUSION

To ensure external validity, the implementation and efficacy of this curriculum should be studied at a variety of outside helicopter emergency medical services organizations to demonstrate its value in a diverse group of learners. Furthermore, the clinical interpretability of sonographs performed by subjects in real clinical settings, as well as their interpretation and clinical actions taken in response to obtained images is crucial to truly determining the practical value of the educational intervention. Finally, the impact of ultrasonography on clinical decision-making by HEMS crews in the out-of-hospital setting should be studied to determine the utility of focusing time and resources on developing skills in ultrasonography in this population. The overall success of the chosen evaluation measures and the confidence of HEMS personnel in performing POCUS after undergoing the novel curriculum suggests that it is both feasible and effective in preparing HEMS crews to competently perform the eFAST exam.

## Supplementary Information







## Figures and Tables

**Table 1:** Kern’s six-step framework for curriculum design.

Kern's Step	POCUS curriculum for critical care personnel
1. Problem identification and general needs assessment	• POCUS can be used to identify and guide treatment for acute, life-threatening pathologies commonly managed by HEMS crews. • There is no universally validated curriculum widely available for teaching POCUS skills to HEMS personnel.
2. Targeted needs assessment	• It is important that the curriculum used to teach the cognitive and psychomotor skills necessary to perform and interpret POCUS is feasible, acceptable, and effective for HEMS crews. • There is little literature on the feasibility and efficacy of curricula for training non-physician field personnel in POCUS.
3. Goals and objectives	• HEMS crew members will be able to describe the clinical indications for the POCUS eFAST exam. • HEMS crew members will be proficient in consistently obtaining clinically interpretable eFAST images using the Philips Lumify ultrasound device. • HEMS crew members will be proficient in interpreting images of the POCUS eFAST and their clinical significance in the context of their critical care practice.
4. Educational strategies	• Four 1-hour lectures focused on foundations of ultrasound concepts and equipment operation, the chest wall ultrasound, the FAST exam, and a review of clinical applications and image review for the combined eFAST exam. • Three formal practice ultrasound sessions were spaced evenly over one year, taught by expert faculty, and using standardized patients. • Unlimited informal practice ultrasound sessions were performed at the convenience of subjects’ schedules with the option of independent practice or practice with an expert instructor. • OSCEs were administered prior to and after completing the didactic and practical curriculum. • Written exams were administered prior to and after completing the didactic and practical curriculum.
5. Implementation	• HEMS crew members participated in pre-curriculum OSCE and written exam to assess baseline knowledge and skills in performing and interpreting the POCUS eFAST. • HEMS crew members were allotted one month to attend lectures live or consume them asynchronously. • Over the course of one year, HEMS crew members participated in 2–3 formal practical educational sessions with expert faculty. • Over the course of one year, HEMS crew members participated in self-paced, informal practice sessions with volunteer patients, independently or with expert faculty, to obtain a minimum of 25 complete eFAST exams. • HEMS crew members participated in post-curriculum OSCE and written exam to assess changes in knowledge and skills in performing and interpreting the POCUS eFAST.
6. Evaluating the effectiveness of the curriculum	• Evaluation of the efficacy of the curriculum with post-implementation OSCE and written exam • Tracking of subjects’ number of clinically interpretable practice ultrasounds • Survey administered after completion of final assessments to all participants to determine curriculum acceptability

*POCUS*, point-of-care-ultrasound; *HEMS*, helicopter emergency medical services; *eFAST*, extended focused assessment with sonography in trauma; *OSCE*, observed structured clinical evaluation.

**Table 2:** Demographics of emergency medical services personnel who participated in ultrasound training.

Age (years)	
Mean	44.2
Median	41.5
Range	28,62
SD	11.9
Gender (n, %)	
Male	11 (68.7)
Female	5 (31.2)
Certification (n, %)	
Paramedic	4 (25)
Registered nurse	10 (62.5)
Both	2 (12.5)
Previous ultrasound training (n, %)	
None	10 (62.5)
1–5 hours	3 (18.8)
5–10 hours	1 (6.3)
>10 hours	0 (0)

*SD*, standard deviation

**Table 3:** Personnel proficiency measures.

	Pre-intervention	Post-intervention	t	p
Written test (%)				
Mean	60.1	82.2	3.41	<0.001
Median	62.7	85		
Range	45.1–88.2	71.1–94.2		
Standard deviation	11.9	8.4		
Personnel with a passing score	12.5	100		
OSCE (%)				
Mean	30.8	95.3		
Median	25.7	95.5	6.87	<0.001
Range	20.0–61.0	83.5–100		
Standard deviation	21.1	5.2		
			X^2^	p
Personnel with >/= 1 critical fail	100	0	21.2	<0.001
Personnel with a passing score	0	100	21.2	<0.001
Practice sonographs				
Mean practice scans performed	27.5			
Mean FAST score	4.73			
Mean chest wall score	5.64			
Mean clinically acceptable FAST scans	21.2			
Mean clinically acceptable chest wall scans	24.4			
			R	R^2^
Increased practice scans and score- FAST			0.44	0.18
Increased practice scans and score- Chest			0.45	0.19
Increased practice scans and interpretable sonographs- FAST			0.86	0.75
Increased practice scans and interpretable sonographs- Chest			0.95	0.91

*OSCE*, observed structured clinical evaluation; *FAST*, focused assessment with sonography in trauma.

**Table 4:** Personnel acceptability ratings (n=11) (n, %).

	Agree/strongly agree	Neutral	Disagree/strongly disagree
The lecture component of the ultrasound curriculum was useful.	10 (90.1)	0 (0)	1 (9.1)
Time spent on ultrasound education made it difficult for me to accomplish my other daily career responsibilities.	2 (18.1)	0 (0)	9 (81.8)
I felt as though the ultrasound curriculum was appropriate for nurses and paramedics.	9 (81.8)	1 (9.1)	1 (9.1)
After this education, I can confidently conduct a lung/chest wall ultrasound exam.	9 (81.8)	1 (9.1)	1 (9.1)
After this education, I can confidently conduct a FAST ultrasound exam.	9 (81.8)	1 (9.1)	1 (9.1)
I feel as though I will be able to maintain my ultrasound skills.	8 (72.7)	1 (9.1)	2 (18.1)
I have gained enough knowledge and experience to comfortably use ultrasound in flight to make clinical decisions as they pertain to lung and FAST ultrasound exams.	8 (72.7)	1 (9.1)	2 (18.1)
I will incorporate my new ultrasound knowledge and skill in the prehospital setting.	10 (90.1)	1 (9.1)	0 (0)
I am satisfied with my participation in ultrasound education.	9 (81.8)	1 (9.1)	1 (9.1)

*FAST*, focused assessment with sonography in trauma.
