# Supplementary material for: A Novel Point-of-care Ultrasound Curriculum for Air Critical Care Personnel
Source: West J Emerg Med. 2023 Jan 9;24(1):30–7. doi: 10.5811/westjem.2022.12.57599 (PMC9897250; doi:10.5811/westjem.2022.12.57599)
Supplement: Supplementary file 1 [file wjem-24-30-s001.pdf]

LifeFlight E-FAST Written Test
Version 1
Please note still images used in this document reflect videos that will be available during exam.
Grading criteria: Passing grade is > 70% correct total with 90% correct from image interpretation section. To be graded by member of ultrasound division.

Image Acquisition

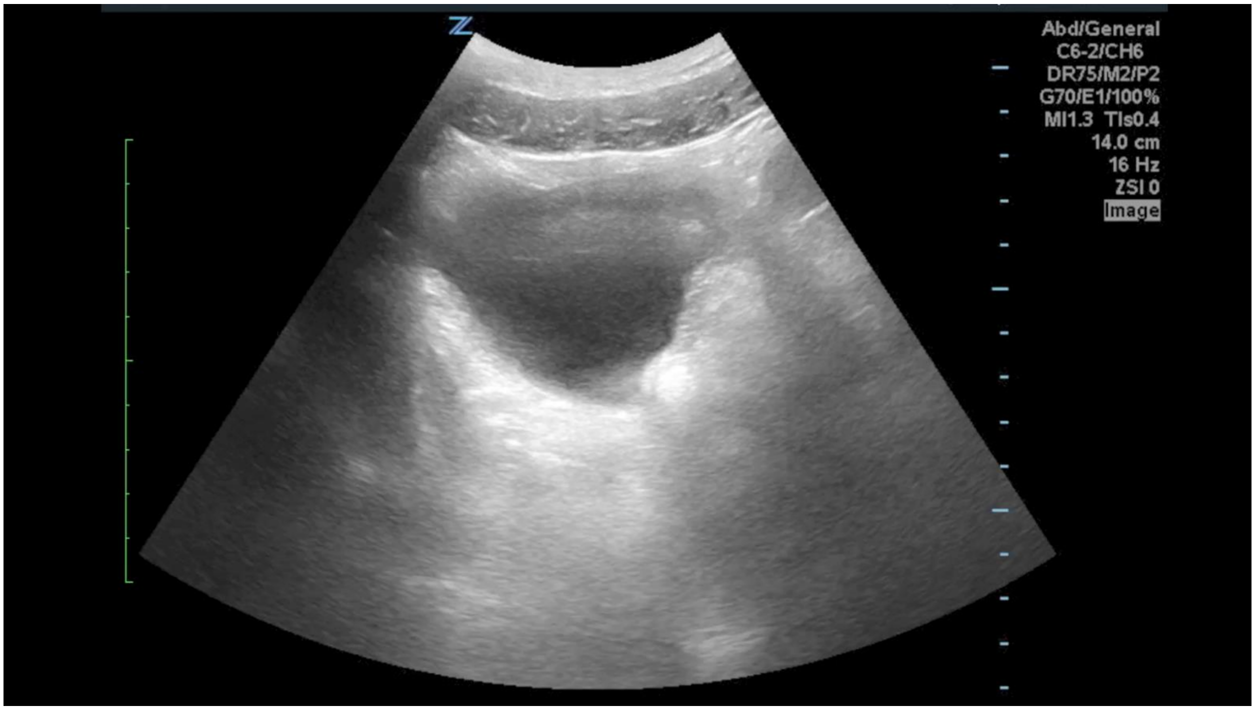

For this image please indicate if the gain is set appropriately, too high or too low. (multiple choice, select 1)

- Appropriate
- **Too high**
- Too low

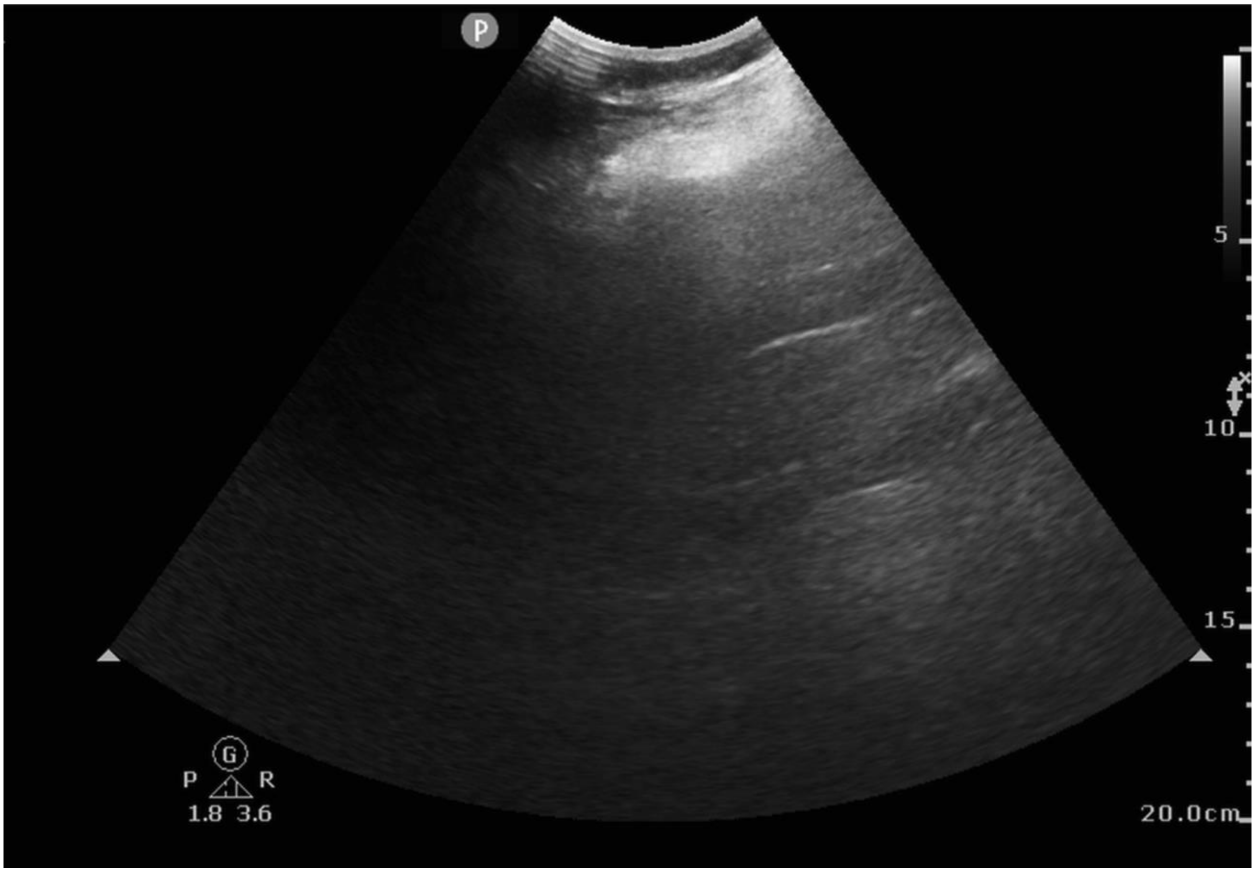

For this image please indicate if the gain is set appropriately, too high or too low. (multiple choice, select 1)

- Appropriate
- Too high
- **Too low**

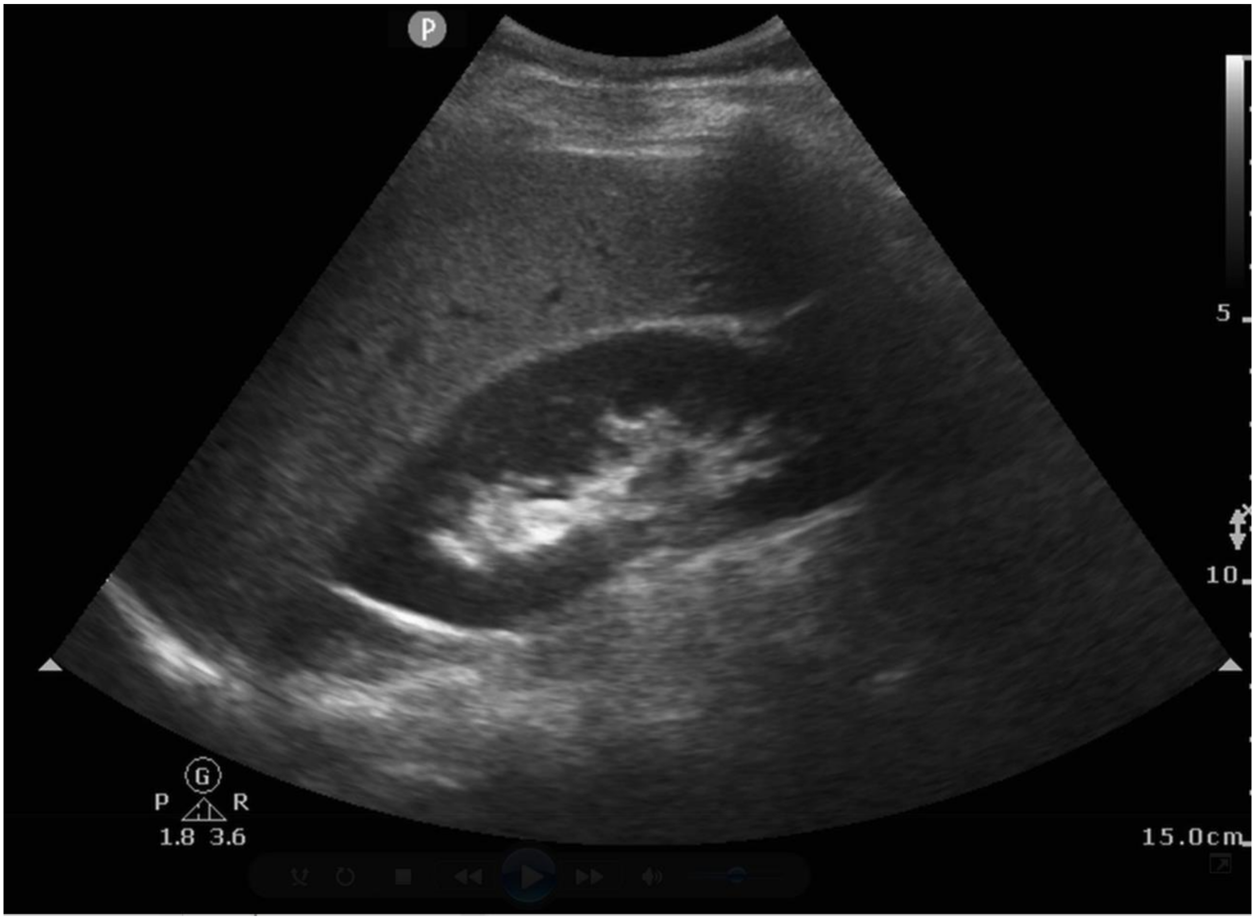

For this image please indicate if the gain is set appropriately, too high or too low. (multiple choice, select 1)

- **Appropriate**
- Too high
- Too low

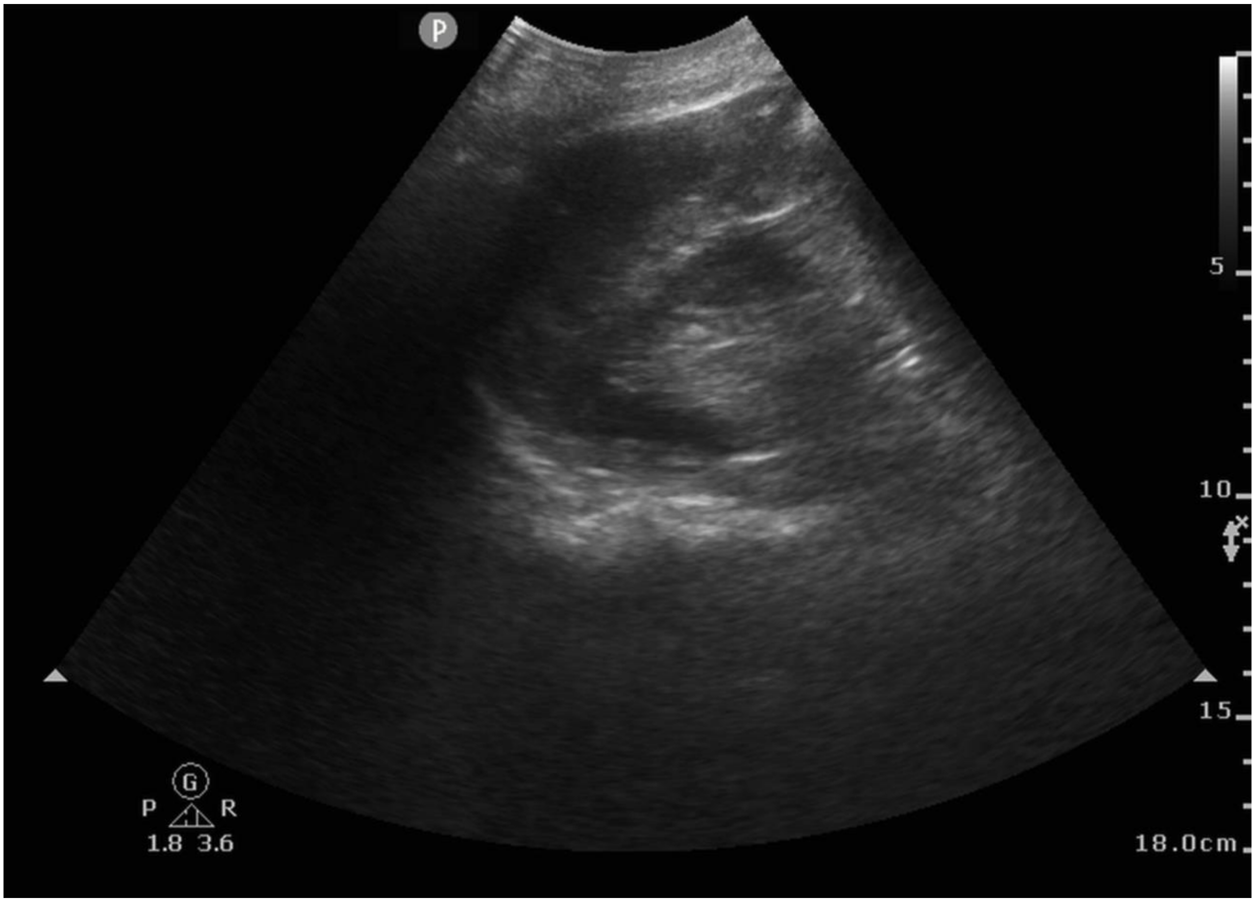

For this image please indicate if the depth is set appropriately, too high or too low. (multiple choice, select 1)

- Appropriate
- **Too high**
- Too low

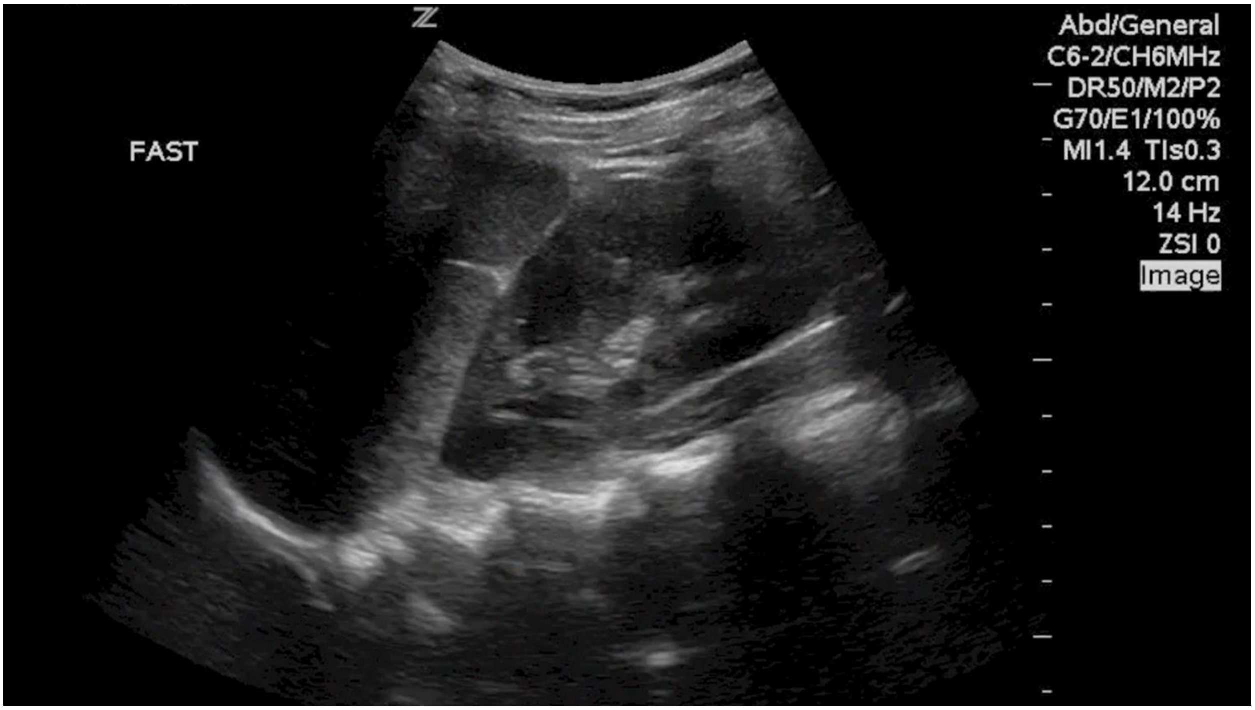

For this image please indicate if the depth is set appropriately, too high or too low. (multiple choice, select 1)

- **Appropriate**
- Too high
- To low

List all views of the E-FAST (free text)

- Chest wall right and left
- Subxiphoid or parasternal long
- Right upper quadrant
- Left upper quadrant
- Bladder (2 views)

On ultrasound, free fluid appears as: (multiple choice, select 1)

- Anechoic (dark)
- Echogenic (bright)

Which probe should be used to perform the E-FAST exam? (multiple choice, select 1)

- Phased array
- Curvilinear

Medical Knowledge

Please list all landmarks visualized in the right upper quadrant view:
(free text)

- Diaphragm
- Morrison's pouch (or hepatorenal space)
- Liver tip
- Inferior pole of kidney

Please list all landmarks visualized in the left upper quadrant view:
(free text)

- Diaphragm
- Splenorenal space
- Spleen tip
- Inferior pole of kidney

In the left upper quadrant, where is free fluid most likely to be seen first? (free text)

- Spleen tip

In the right upper quadrant, where is free fluid most likely to be seen first? (free text)

- Either morrison's pouch or hepatorenal space

If the spine is visualized cranial (above) the diaphragm, is this: (multiple choice select 1)

- Normal
- Indicates fluid in the thorax
- Indicates pneumothorax

In the bladder view, free fluid is most likely to appear: (multiple choice select 1)

- Inferior to (below) the bladder
- On either side of the bladder
- Above the bladder

What additional mode can be used to help identify the presence or absence of lung sliding?

- (fill in) M-mode
    - Using this mode, what would a barcode sign indicate? (fill in) pneumothorax
    - Using this mode, what would a seashore sign indicate? (fill in) normal

List two medical conditions which can produce free fluid in the abdomen or chest in the absence of trauma and hemorrhage. (free text)

- Congestive heart failure
- Liver disease or ascites
- Metastatic disease
- Empyema
- Lymphoma
- Cancer
- Pulmonary embolism

- Tuberculosis
- Systemic lupus erythematosus
- Nephrotic syndrome
- Pericarditis

List 3 things that differentiate a pericardial effusion from a normal fat pad (free text)

- hypoechoic (gray)
- Does not significantly change shape or form with cardiac cycle
- Does not wrap around heart

Image Interpretation

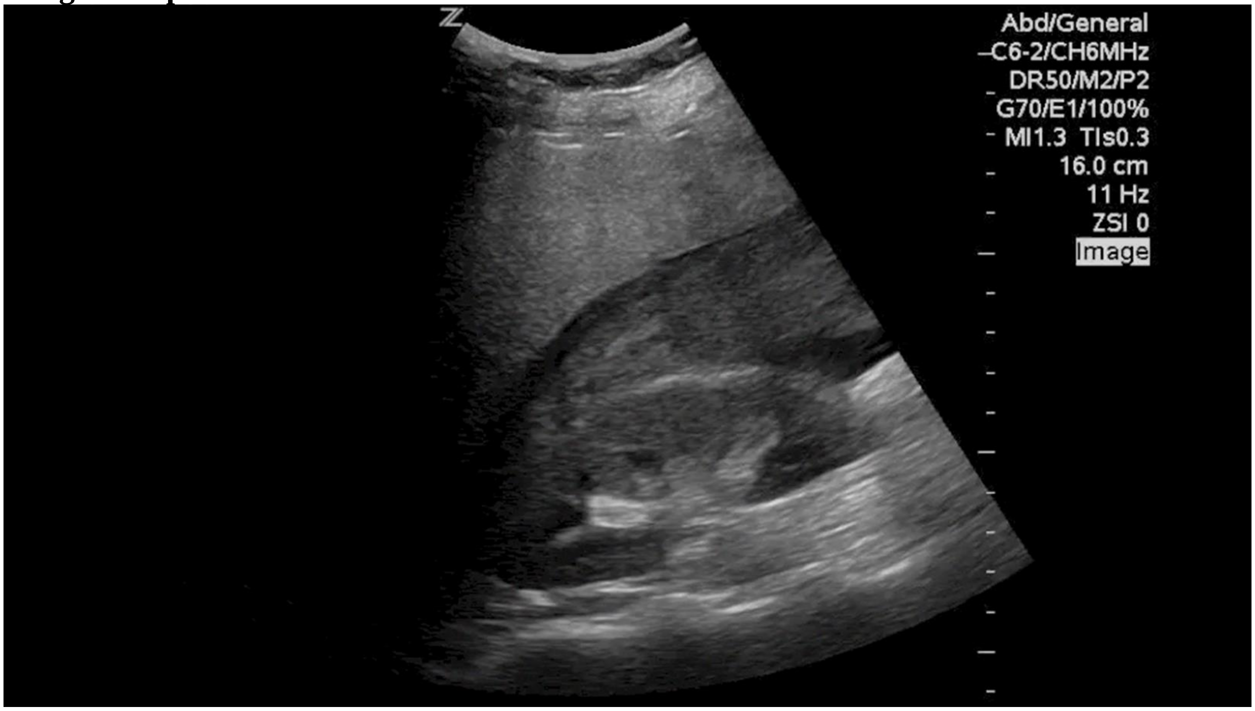

What view is this? (multiple choice select 1)

- Chest wall
- Subxyphoid
- Parasternal long axis
- **Right upper quadrant**
- Left upper quadrant
- Bladder

What is the finding? (multiple choice select 1)

- Positive for pneumothorax
- Positive for free fluid in the chest
- Positive for pericardial effusion
- **Positive for free fluid in the abdomen**
- None of these

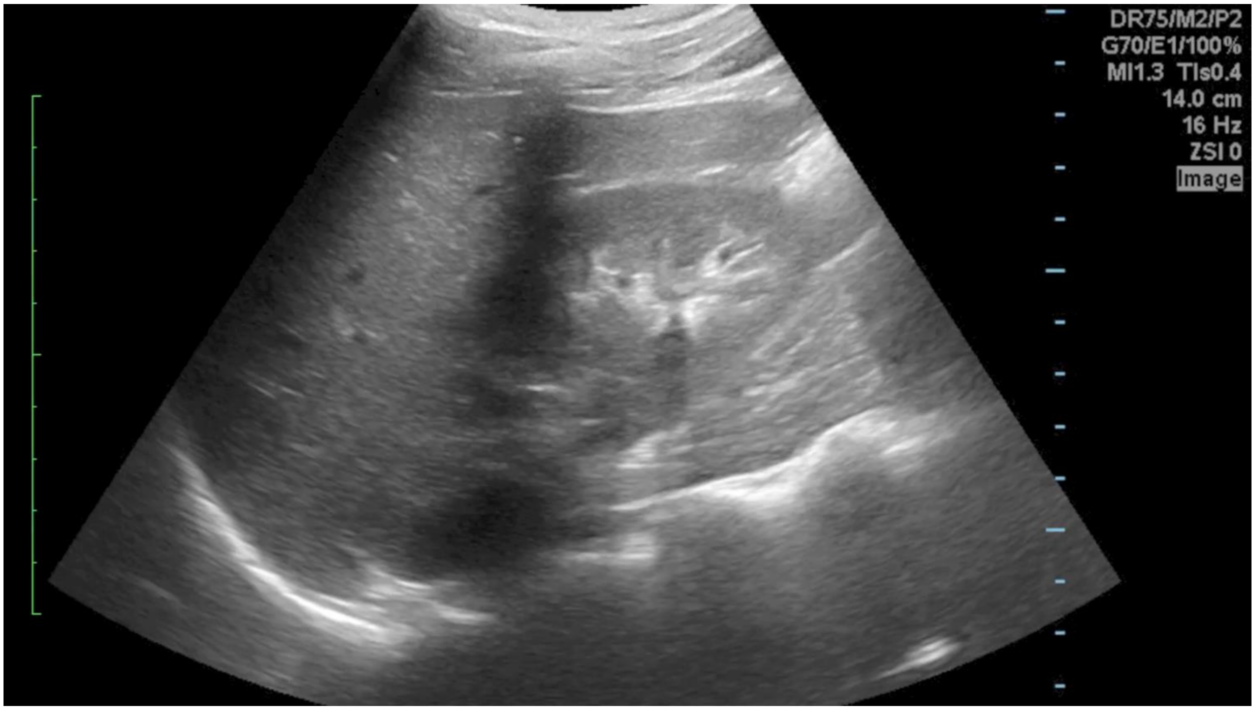

What view is this? (multiple choice select 1)

- Chest wall
- Subxiphoid
- Parasternal long axis
- **Right upper quadrant**
- Left upper quadrant
- Bladder

What is the finding? (multiple choice select 1)

- Positive for pneumothorax
- Positive for free fluid in the chest
- Positive for pericardial effusion
- Positive for free fluid in the abdomen
- **None of these**

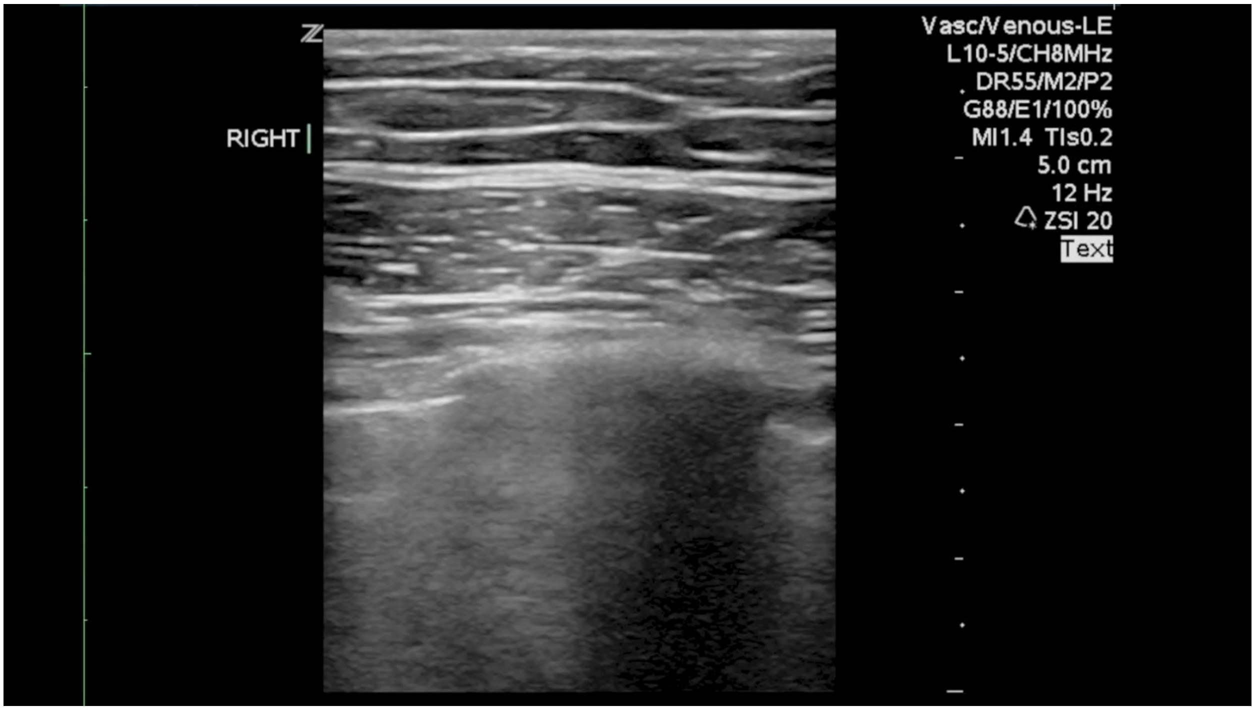

What view is this? (multiple choice select 1)

- **Chest wall**
- Subxiphoid
- Parasternal long axis
- Right upper quadrant
- Left upper quadrant
- Bladder

What is the finding? (multiple choice select 1)

- Positive for pneumothorax
- Positive for free fluid in the chest
- Positive for pericardial effusion
- Positive for free fluid in the abdomen
- **None of these**

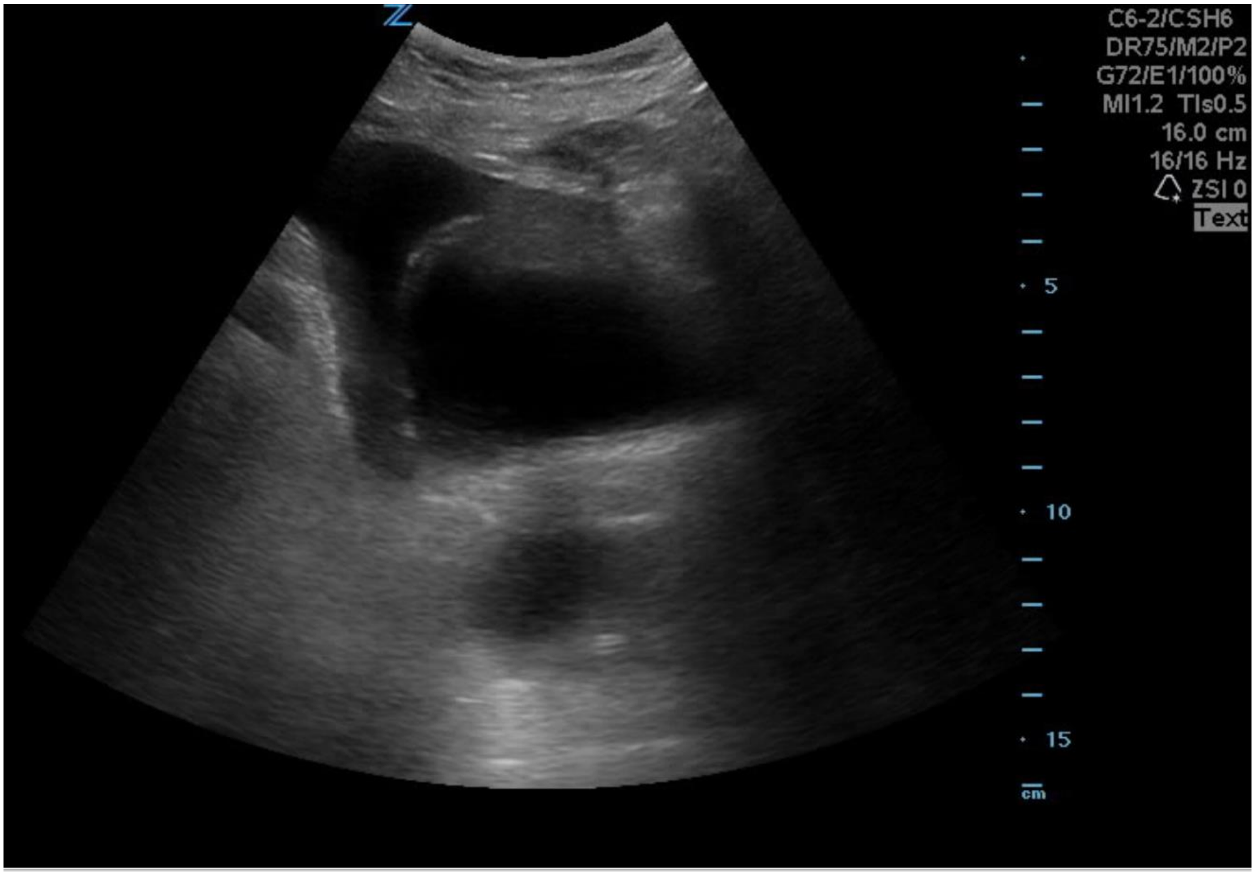

What view is this? (multiple choice select 1)

- Chest wall
- Subxiphoid
- Parasternal long axis
- Right upper quadrant
- Left upper quadrant
- **Bladder**

What is the finding? (multiple choice select 1)

- Positive for pneumothorax
- Positive for free fluid in the chest
- Positive for pericardial effusion
- **Positive for free fluid in the abdomen**
- None of these

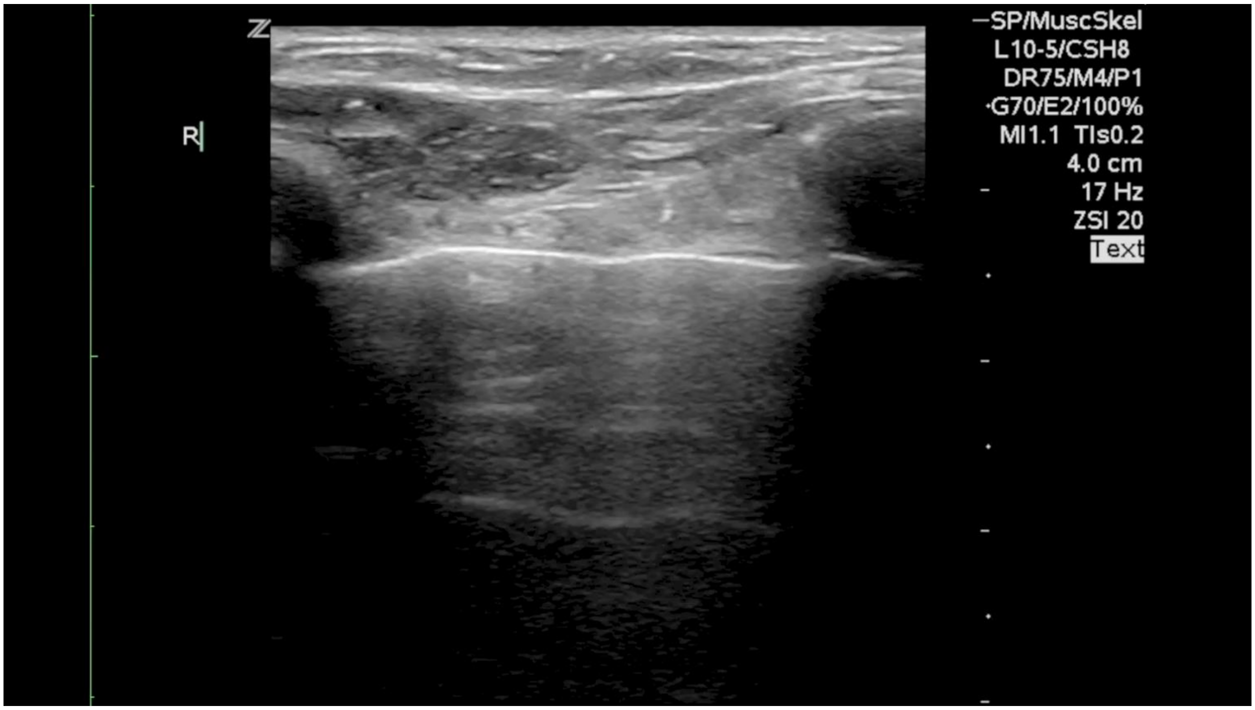

What view is this? (multiple choice select 1)

- **Chest wall**
- Subxiphoid
- Parasternal long axis
- Right upper quadrant
- Left upper quadrant
- Bladder

What is the finding? (multiple choice select 1)

- **Positive for pneumothorax**
- Positive for free fluid in the chest
- Positive for pericardial effusion
- Positive for free fluid in the abdomen
- None of these

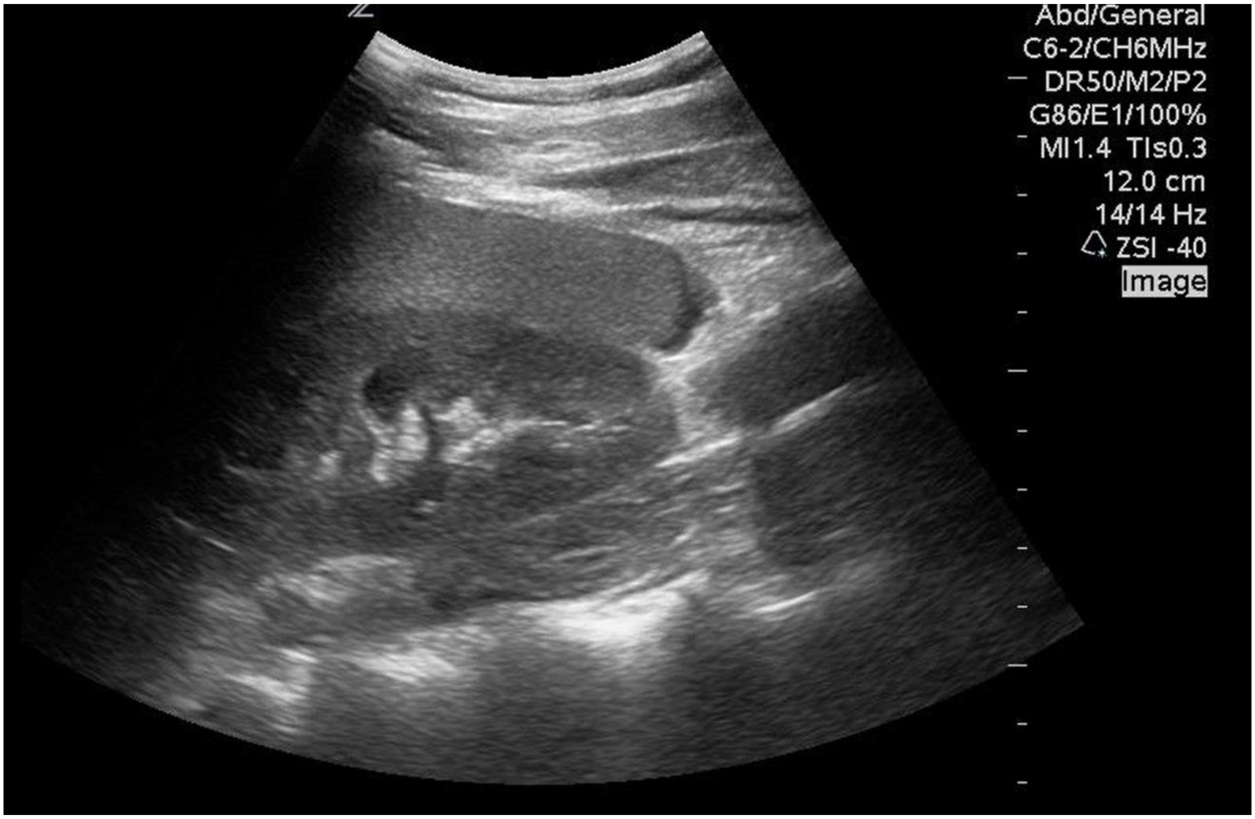

What view is this? (multiple choice select 1)

- Chest wall
- Subxiphoid
- Parasternal long axis
- Right upper quadrant
- **Left upper quadrant**
- Bladder

What is the finding? (multiple choice select 1)

- Positive for pneumothorax
- Positive for free fluid in the chest
- Positive for pericardial effusion
- **Positive for free fluid in the abdomen**
- None of these

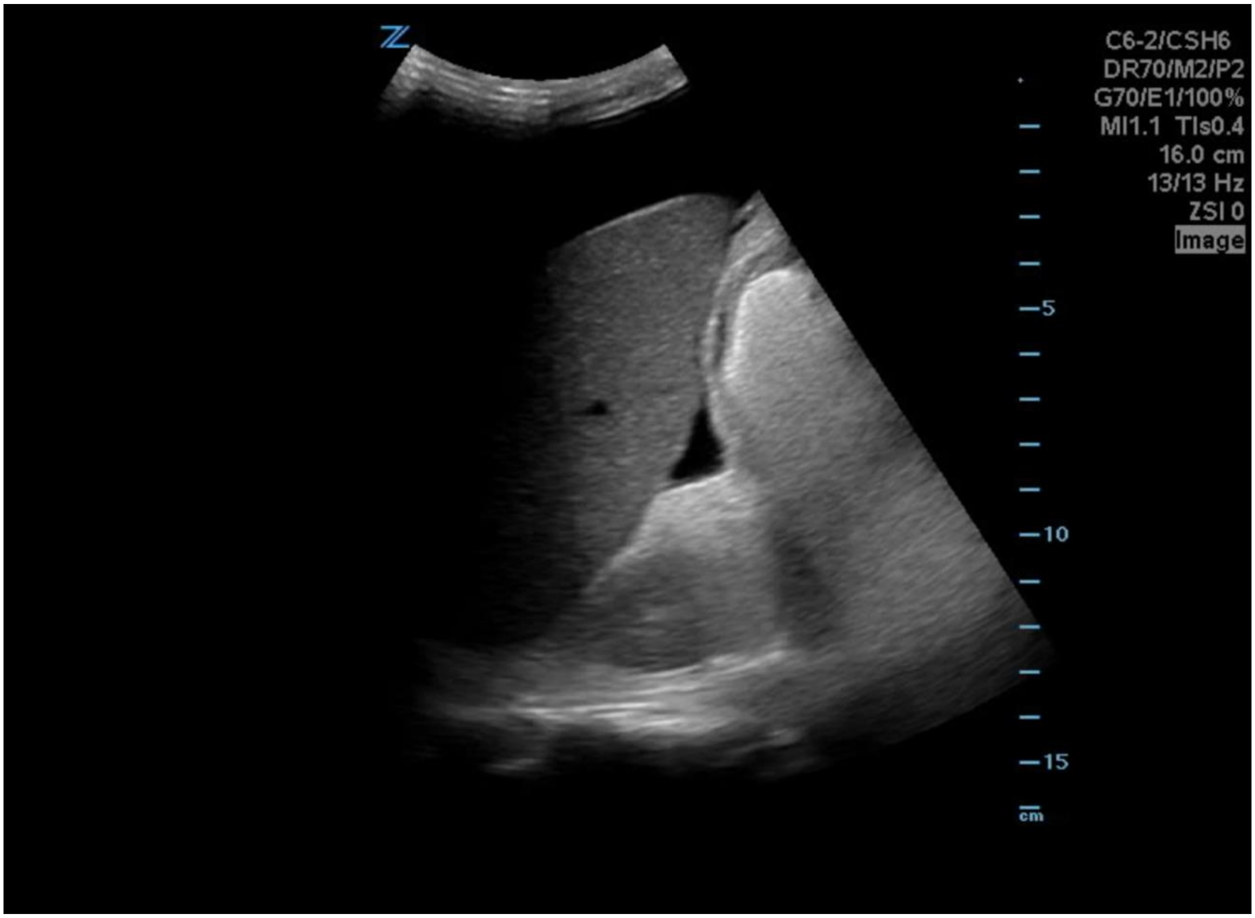

What view is this? (multiple choice select 1)

- Chest wall
- Subxiphoid
- Parasternal long axis
- **Right upper quadrant**
- Left upper quadrant
- Bladder

What is the finding? (multiple choice select 1)

- Positive for pneumothorax
- Positive for free fluid in the chest
- Positive for pericardial effusion
- **Positive for free fluid in the abdomen**
- None of these

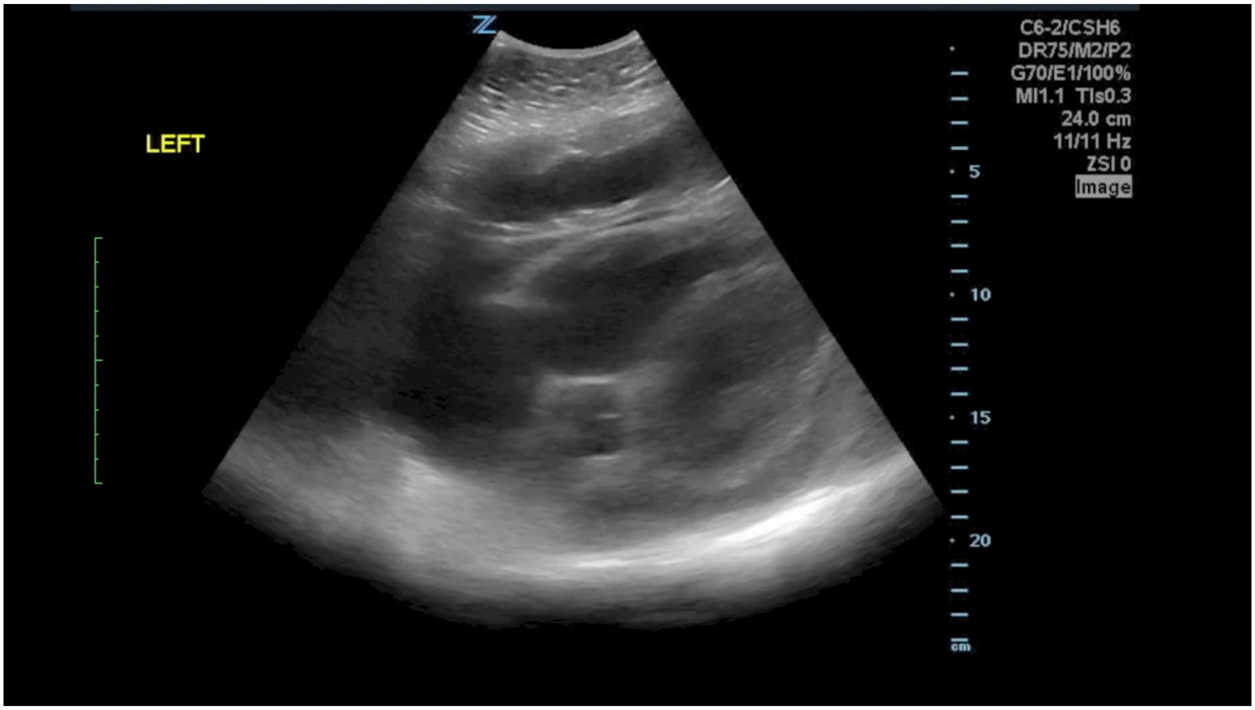

What view is this? (multiple choice select 1)

- Chest wall
- **Subxiphoid**
- Parasternal long axis
- Right upper quadrant
- Left upper quadrant
- Bladder

What is the finding? (multiple choice select 1)

- Positive for pneumothorax
- Positive for free fluid in the chest
- **Positive for pericardial effusion**
- Positive for free fluid in the abdomen
- None of these

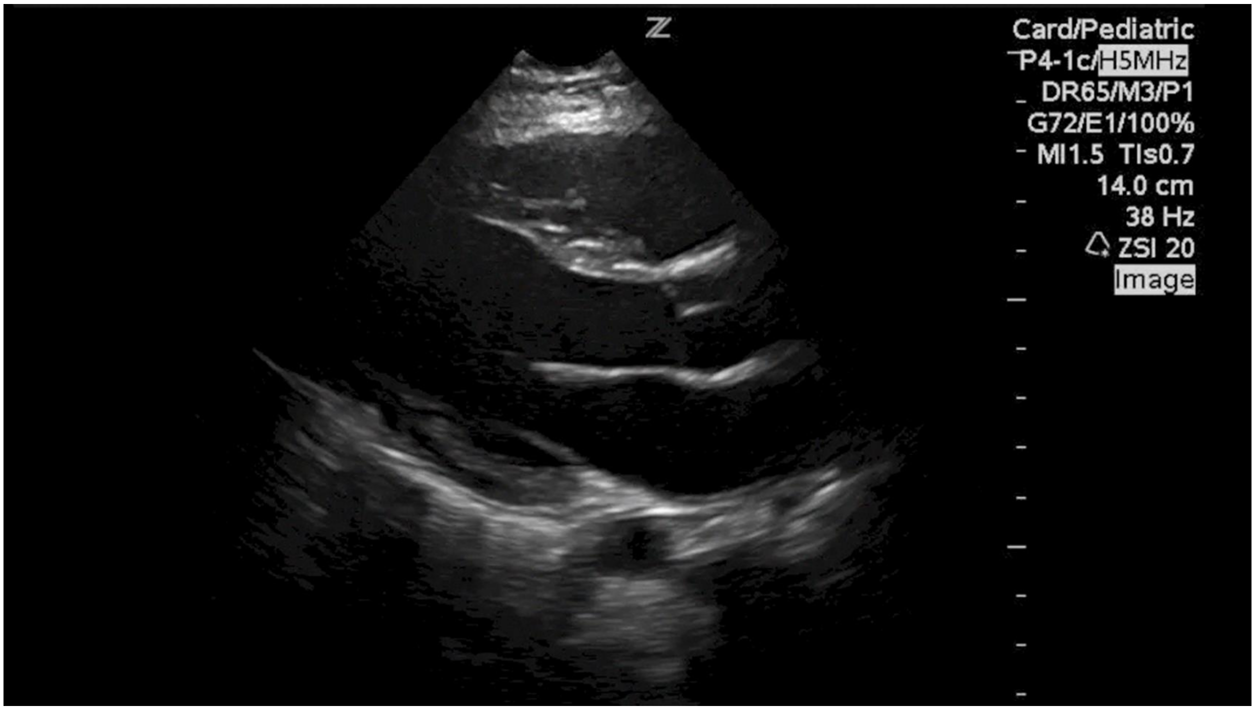

What view is this? (multiple choice select 1)

- Chest wall
- Subxiphoid
- **Parasternal long axis**
- Right upper quadrant
- Left upper quadrant
- Bladder

What is the finding? (multiple choice select 1)

- Positive for pneumothorax
- Positive for free fluid in the chest
- Positive for pericardial effusion
- Positive for free fluid in the abdomen
- **None of these**

Which probe was used to obtain this image (fill in)
Phased array

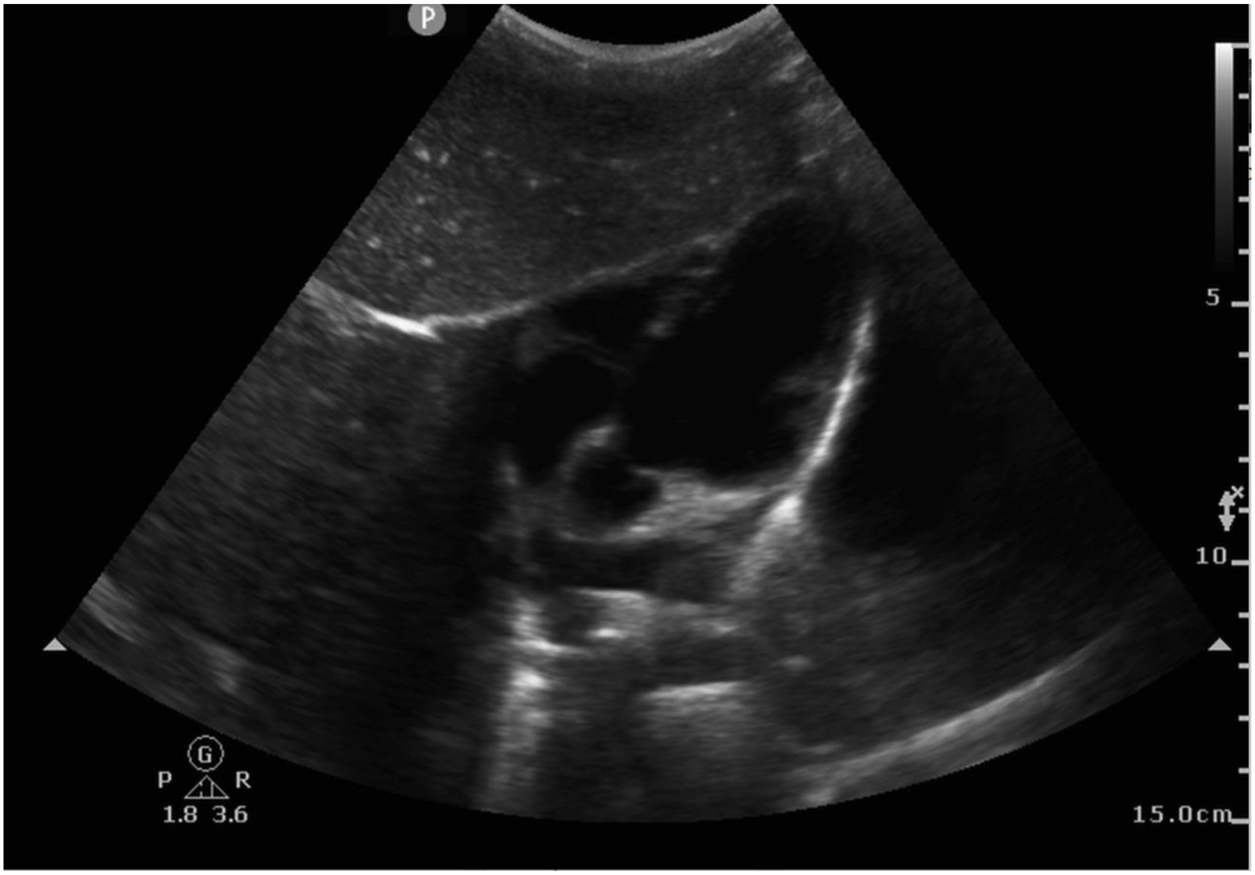

What view is this? (multiple choice select 1)

- Chest wall
- **Subxiphoid**
- Parasternal long axis
- Right upper quadrant
- Left upper quadrant
- Bladder

What is the finding? (multiple choice select 1)

- Positive for pneumothorax
- Positive for free fluid in the chest
- Positive for pericardial effusion
- Positive for free fluid in the abdomen
- **None of these**
