# Supplementary material for: A Novel Point-of-care Ultrasound Curriculum for Air Critical Care Personnel
Source: West J Emerg Med. 2023 Jan 9;24(1):30–7. doi: 10.5811/westjem.2022.12.57599 (PMC9897250; doi:10.5811/westjem.2022.12.57599)
Supplement: Supplementary file 2 [file wjem-24-30-s002.pdf]

**Lung, right**	yes	no
**Pleura visualized**		
Visualize at least two ribs in cross section with pleura between		
**Depth is sufficient to visualize pleura**		
There is between 1 cm and 4 cm of depth beyond the pleura		
Record B-Mode Image		
No probe movement during recording		
Gain set to appropriate level		
**Gain is set such that pleural movement could be visualized (not so high or so low that image is not interpretable)**		
Activate M-Mode and move cursor to be over pleura		
Record M-Mode image		
**Correctly interpret lung sliding/no lung sliding in B-Mode**		
**Correctly interpret lung sliding/no lung sliding in M-Mode**		
**Correctly verbalize the clinical interpretation of the study based on lung sliding (positive or negative for pneumothorax)**		

**Lung, left**	yes	no
**Pleura visualized**		
Visualize at least two ribs in cross section with pleura between		
**Depth is sufficient to visualize pleura**		
There is between 1 cm and 4 cm of depth beyond the pleura		
Record B-Mode Image		
No probe movement during recording		
Gain set to appropriate level		
**Gain is set such that pleural movement could be visualized (not so high or so low that image is not interpretable)**		
Activate M-Mode and move cursor to be over pleura		
Record M-Mode image		
**Correctly interpret lung sliding/no lung sliding in B-Mode**		
**Correctly interpret lung sliding/no lung sliding in M-Mode**		
**Correctly verbalize the clinical interpretation of the study based on lung sliding (positive or negative for pneumothorax)**		

**Cardiac**	yes	no
Subxyphoid view attempted first		
Parasternal view attempted if subxyphoid view unable to obtain (leave blank if n/a)		
**Subxyphoid view or parasternal long axis view obtained and recorded**		
Adequate depth with > 1 cm imaged beyond posterior pericardium		
There is adequate gain to visualize pericardium		
**Correctly interpret presence or absence of pericardial effusion**		

**RUQ**	yes	no
**RUQ view obtained and recorded**		
Indicator oriented towards patient's head		
Visualize liver tip and show to examiner		
**Visualize Morison's pouch and show to examiner**		
Scan through Morison's pouch		
Visualize inferior pole of kidney and show to examiner		
**Identify diaphragm and show to examiner**		
There is adequate depth to visualize the diaphragm and potential pathology behind (such as spine sign)		
Depth is set such that there is less than 25% empty space in the far field		
Gain is appropriate		
**Gain is neither so high or so low that image is not interpretable**		
**Verbalize presence or absence of anechoic material in Morison's pouch, at the liver tip and inferior renal pole (if visualized)**		
**Verbalize presence or absence of anechoic fluid in the thorax**		
**Interpret above findings correctly as positive or negative**		

**LUQ**	yes	no
**LUQ view obtained and recorded**		
Indicator oriented towards patient's head		
**Visualize spleen tip and show to examiner**		
Visualize splenorenal space and show to examiner		
Scan through splenorenal space.		
Visualize inferior pole of kidney and show to examiner		
**Identify diaphragm and show to examiner**		
There is adequate depth to visualize the diaphragm and potential pathology behind (such as spine sign)		
Depth is set such that there is less than 25% empty space in the far field		
Gain is appropriate		
**Gain is neither so high or so low that image is not interpretable**		
**Verbalize presence or absence of anechoic material at the spleen tip, the splenorenal space and inferior renal pole (if visualized)**		
**Verbalize presence or absence of anechoic fluid in the thorax**		
**Interpret above findings correctly as positive or negative**		

**Suprapubic**	yes	no
**Two views of the bladder obtained and recorded**		
Dynamic images recorded fully sweeping through the bladder in 2 views		
Depth is appropriate with at least 2 cm visualized behind bladder		
Gain is appropriate that area behind bladder is not overgained		
**Gain is such that image is interpretable**		

Area behind bladder identified and shown to examiner		
**Verbalize presence or absence of anechoic material behind the bladder**		
**Correctly identify free fluid or no free fluid in pelvis**		
Iliac vessels misidentified as free fluid (-1 point if yes)		
Bowel misidentified as free fluid (-1 point if yes)		
