# Supplementary material for: A Novel Point-of-care Ultrasound Curriculum for Air Critical Care Personnel
Source: West J Emerg Med. 2023 Jan 9;24(1):30–7. doi: 10.5811/westjem.2022.12.57599 (PMC9897250; doi:10.5811/westjem.2022.12.57599)
Supplement: Supplementary file 3 [file wjem-24-30-s003.pdf]

appendix c: scoring for eFAST exam

1. possible score ratings assigned to practice ultrasound scans

Score	Indication
8	perfect technique
7	excellent technique on all views
6	excellent technique on most views
5	good technique on most views - images clear
4	adequate technique - images interpretable
3	adequate technique - images atypical
2	poor technique - not all views interpretable
1	poor technique - no images interpretable

2. Example eFAST Interpretation and scoring rubric

2.1 Chest Wall Ultrasound

Type: Chest Wall Ultrasound

```
Overall Technical Rating (1 to 8): 7 – excellent technique on all views
```

Your Readings	We agreed
primary<br>○ (+) Pneumothorax<br>◉ (-) Pneumothorax<br>○ ? Pneumothorax<br>○ No reading<br>○ Abnormal<br>secondary<br>☐ (+) effusion<br>☐ (+) rib fx<br>☐ Thoracic fluid visualized<br>☐ Pericardial effusion visualized<br>☐ Abnormal pulmonary parenchyma<br>advanced	

Image Ratings:

	Left Chest wall	Right Chest wall
	☑ No Errors	☑ No Errors
**Images/ Image Elements:**	☐ Image Missing<br>☐ multiple images obtained<br>☐ M Mode Images obtained	☐ Image Missing<br>☐ multiple images obtained<br>☐ M Mode Images obtained
**Machine Settings:**	☐ Depth too deep<br>☐ Depth too shallow<br>☐ Too much gain<br>☐ Too little gain	☐ Depth too deep<br>☐ Depth too shallow<br>☐ Too much gain<br>☐ Too little gain
**Probe Mechanics:**	☐ Image Blurry (motion artifact)<br>☐ Image too caudal (view of cardiac space)	☐ Image Blurry (motion artifact)<br>☐ Image too caudal (view of cardiac space)

2.2 Fast Exam

Type: FAST

Overall Technical Rating (1 to 8): 8 - perfect technique

Your Readings **We agreed**

primary

- ( ) (+) Free Fluid
- (•) (-) Free Fluid
- ( ) ? Free fluid
- ( ) No reading
- ( ) Abnormal

secondary

- [ ] Renal cyst
- [ ] Intrauterine pregnancy
- [ ] Thoracic Fluid
- [ ] Bladder Clot
- [ ] Bladder Mass

advanced

- [ ] Solid organ injury

Image Ratings:

	Sub-Xyphoid	RUQ	LUQ	Supra-pubic
	[x] No Errors	[x] No Errors	[x] No Errors	[x] No Errors
**Images/ Image Elements:**	[ ] Image missing<br>[ ] Incomplete view of heart<br>[ ] Pericardial Space Indistinct<br>[ ] Parasternal long view obtained instead	[ ] Image missing<br>[ ] Centered on kidney and not on Morrison's Pouch<br>[ ] Diaphragm not visualized<br>[ ] Inferior renal tip or liver margin not visualized	[ ] Image missing<br>[ ] Centered on kidney and not on kidney-spleen interface<br>[ ] Diaphragm not visualized<br>[ ] Inferior renal tip or spleen tip not visualized	[ ] Image missing<br>[ ] Only one view of bladder included<br>[ ] Bladder decompressed by foley
**Machine Settings:**	[ ] Depth set too deep<br>[ ] Depth set too shallow<br>[ ] Too much gain<br>[ ] Too little gain	[ ] Depth set too deep<br>[ ] Depth set too shallow<br>[ ] Too much gain<br>[ ] Too little gain	[ ] Depth set too deep<br>[ ] Depth set too shallow<br>[ ] Too much gain<br>[ ] Too little gain	[ ] Depth set too deep<br>[ ] Depth set too shallow<br>[ ] Too much gain<br>[ ] Too little gain
**Probe Mechanics:**	[ ] Orientation flipped<br>[ ] Oblique orientation<br>[ ] Motion artifact - image blurry	[ ] Orientation flipped<br>[ ] Oblique orientation<br>[ ] Probe too anterior<br>[ ] Probe too posterior<br>[ ] Motion artifact - image blurry	[ ] Orientation flipped<br>[ ] Oblique orientation<br>[ ] Probe too anterior<br>[ ] Motion artifact - image blurry	[ ] Orientation flipped<br>[ ] Oblique orientation<br>[ ] Probe too superior<br>[ ] Motion artifact - image blurry
